# Integrated analysis of transcriptome and genome variations in pediatric T cell acute lymphoblastic leukemia: data from north Indian tertiary care center

**DOI:** 10.1186/s12885-024-12063-6

**Published:** 2024-03-08

**Authors:** Minu Singh, Pankaj Sharma, Prateek Bhatia, Amita Trehan, Rozy Thakur, Sreejesh Sreedharanunni

**Affiliations:** 1 Haematology-Oncology Unit, Department of Paediatrics, Postgraduate Institute of Medical Education and Research, Sector -12, 160012 Chandigarh, India; 2 Department of Haematology, Postgraduate Institute of Medical Education and Research, Chandigarh, India

**Keywords:** T cell acute lymphoblastic leukemia, Transcriptome, Exome sequencing, Gene fusion

## Abstract

**Introduction:**

T-cell acute lymphoblastic leukemia (T-ALL) is a genetically heterogeneous disease with poor prognosis and inferior outcome. Although multiple studies have been perform on genomics of T-ALL, data from Indian sub-continent is scarce.

**Methods:**

In the current study we aimed to identify the genetic variability of T-ALL in an Indian cohort of pediatric (age ≤ 12 years) T-ALL patients (*n* = 25) by whole transcriptome sequencing along with whole exome sequencing and correlated the findings with clinical characteristics and disease outcome.

**Results:**

The median age was 7 years (range 3 -12 years). RNA sequencing revealed a definitive fusion event in 14 cases (56%) (including a novel fusions) with *STIL::TAL1* in 4 (16%), followed by *NUP21::ABL1, TCF7::SPI1, ETV6::HDAC8, LMO1::RIC3, DIAPH1::JAK2, SETD2::CCDC12* and *RCBTB2::LPAR6* in 1 (4%) case each. Significant aberrant expression was noted in *RAG1* (64%), *RAG2* (80%), *MYCN* (52%), *NKX3-1* (52%),* NKX3-2* (32%), *TLX3 *(28%), *LMO1* (20%) and *MYB* (16%) genes. WES data showed frequent mutations in *NOTCH1* (35%) followed by *WT1* (23%), *FBXW7* (12%), *KRAS* (12%), *PHF6* (12%) and *JAK3* (12%). Nearly 88.2% of cases showed a deletion of *CDKN2A/CDKN2B/MTAP* genes. Clinically significant association of a better EFS and OS (*p*=0.01) was noted with *RAG2* over-expression at a median follow up of 22 months, while a poor EFS (*p*=0.041) and high relapse rate (*p*=0.045) was observed with *MYB* over-expression.

**Conclusion:**

Overall, the present study demonstrates the frequencies of transcriptomic and genetic alterations from Indian cohort of pediatric T-ALL and is a salient addition to current genomics data sets available in T-ALL.

**Supplementary Information:**

The online version contains supplementary material available at 10.1186/s12885-024-12063-6.

## Introduction

T-cell acute lymphoblastic leukemia (T-ALL) is a highly aggressive form of ALL and represents 15–20% of pediatric ALL cases [1]. Even with highly intensified therapy, 25% of T-ALL patients experience relapse and have lower post-relapse survival compared to the B-lineage ALL [1]. The genetic heterogeneity of the disease makes T-ALL risk stratification difficult and hence all cases are treated upfront as high-risk with intensified therapy regimen. The high dose multi-agent chemotherapy is often associated with severe toxicities and long-term side effects. Thus, improving understanding of T-ALL biology through the identification and characterization of carcinogenic lesions is essential for better prognostic classification and treatment of the disease.

With the advent of next-generation sequencing techniques, many genetic abnormalities have been found in T-ALL over the last few decades. Aberrant expression of genes such as *LMO1, LMO2, TAL1, TLX1, TLX3, NKX2-1* and other transcription factors (TFs) have long been known [2]. Whole-exome sequencing (WES), and RNA sequencing (RNA-seq) have extended the list of genetic abnormalities in T-ALL [3, 4]. Besides aberrant expression that constitutes about 40–50% T-ALL [5], RNA seq data has expanded the fusion gene list (30–40%) in T-ALL. Such fusion transcripts can either generate an over-expressing protein as in the case of *TAL1* as a result of *STIL::TAL1* fusion [6, 7] or lead to over-expression of two truncated peptides such as *SET*&*NUP214* in *SET:: NUP214* fusion [8]. A number a novel fusion transcripts have been also identified in studies from different cohorts such as *ZBTB16::ABL1, RCBTB2::LPAR6, DLEU2::SPRYD7, TRAC::SOX8* etc. [9, 10]. Further, in a holistic approach WES along with RNA seq has identified number of gene abnormalities in pathways regulating differentiation, proliferation, self-renewal, and survival of T-cell precursors. High mutation frequencies such as *NOTCH1, JAK-STAT, PI3K-AKT* or *RAS-MAPK* pathway genes have been noted in multiple studies although the frequency varies among cohorts and population being adult or pediatric [11, 12]. Moreover, copy number variations (CNVs), especially high frequency of *CDKN2A/CDKN2B* deletions have been consistently shown across multiple studies [13–15].

Thus, the complex interplay of gene fusions, sequence aberrations and transcriptional expression profiles needs to be increasingly investigated in different cohorts to further refine current models of T-cell leukemia and to identify potential new biomarkers and therapeutic targets. In the current study, RNA-seq and WES analyses were performed in a prospective cohort of pediatric T-ALL cases. A number of rare gene fusions, mutations, aberrant transcripts, and CNVs were identified. Overall, our results point to the need for further large-scale genomic studies to improve patient stratification and optimize treatment strategies for pediatric T-ALL, especially in relation to our distinctly ethnic sub-continental population.

## Materials and methods

### Patients and samples

Newly diagnosed pediatric T-ALL cases (age ≤12 years) confirmed on morphology and immunophenotype (flow cytometry) were enrolled for the study. Cases were classified immunophenotypically by flow cytometry into immature (pro T- and pre T-), cortical and mature T-ALL based on the EGIL criteria [16]. ETP-ALL was recognized based on the previously defined criteria [17]. Complete immunophenotype data in 3 patients was unavailable. Patients were considered as good prednisolone responder at day 8 if absolute blast counts (ABC) were < 1000/ul and poor prednisolone responder if ABC > 1000/ul. Day 8 ABC data was unavailable in6 cases. Patients were treated and followed up uniformly as per the ICiCLe treatment protocol (Clinical Trials Registry-India number, CTRI/2015/12/006,434) [18]. Written informed consent in agreement with the Declaration of Helsinki was taken from children and or guardians and the study was approved by the Institutional Ethics board.

### RNA sequencing

RNA was extracted from PBMCs isolated from patient blood/bone marrow samples by the RNA blood kit (Qiagen Inc.) as per manufacturer’s protocol. NEBNext RNA Ultra II directional protocol was used to prepare the libraries for total RNA. Paired-end whole transcriptome sequencing was performed on the Illumina NovaSeq to generate 60 M, 2 × 150 bp reads/sample. The raw reads were filtered using Trimmomatic for quality scores and adapters. Filtered reads were aligned to Human genome (hg19) using splice aware aligner HISAT2 to quantify reads mapped to each transcript. Alignment percentage of reads were in the range of 91.7–97.5%. Total number of uniquely mapped reads were counted using feature counts. The uniquely mapped reads were then subjected to differential gene expression using Deseq2 (supplementary data- TALL Deseq2 data).

### Gene fusion analysis

The gene fusion studies of allsamples were carried out by detecting fusion events using two different prediction tools namely, FusionCatcher [19] and STARFusion [20]. The read alignment obtained from the above tools were considered for the event prediction. The tools provide both junction and spanning reads from the mapped bam file. The mapped reads from two different tools were considered as best hit. True fusions typically form from exon-exon fusion. The genomic coordinates were checked to ensure they were identical across tools with minimum distance between 5`gene and 3`gene. Manual review was applied to generate the final fusion gene list. Fusions were only considered for further analysis, if they were called by both the callers with at least 5 reads and were not detected in control samples. Novelty of fusions were checked on Mitelman Database of Chromosome Aberrations and Gene Fusions, and ChimerDB [21, 22].

Novel fusion validation: Novel fusion (*ETV6::HDAC8*) identified was validated by qRT-PCR using forward primer TCTATACACACACAGCCGGA and reverse primer CCCTGCAGTCACAAATTCCA.

### Whole exome sequencing

DNA was extracted from PBMCs isolated from blood/bone marrow samples using Qiagen DNA blood mini kit (Qiagen Inc.). The libraries were prepared by standard protocol of Illumina platform. Paired-end sequencing (2 × 101 bp read length) was performed using the Illumina HiSeq 2000/2500 platform. Exome sequencing analysis was performed using Dragen server (Illumina Inc.). The fastq files after demultiplexing were first aligned to reference genome and then the output Sam files were converted to bam. The bam file was then sorted followed by duplicate removal, realignment and re-calibration. Variant caller based on haplotypecaller of GATK was used to generate the variant call files (vcf). VCF files were then uploaded on GeneYX tool [23] for variant identifications, annotations and subsequent reporting.

### Outcome assessment and statistical analysis

Treatment outcome parameters analyzed included relapse free survival (RFS) or relapse rate (RR) - defined as time period from onset of therapy to disease relapse for those achieving complete remission with censoring at death in remission or last contact. Overall survival (OS)- defined as time period from onset of therapy to death with censoring at last contact. Event free survival (EFS)- defined as time period from onset of therapy to any event (relapse/death/abandonment of treatment against medical advice) with censoring at the time of event or last contact. Continuous variables were represented as mean/median (range) and categorical variables as ratio/proportion. Chi-square test was performed for categorical variables between different clinical, hematological and treatment outcome parameters and genetic events. Survival curves (EFS, RR, OS) for overall cohort in relation to different genetic aberrations were calculated using Kaplan Meier curve and log-rank tests. A p-value of < 0.05 was considered as significant. All statistical analysis was performed using SPSS v26.0.

## Results

### Clinical characteristics of the patients

Twenty five cases of newly diagnosed pediatric T-ALL were enrolled for the study. The median age of the patients at diagnosis was 7 years (range 3–12 years) with male to female ratio of 11.5:1. The median WBC count was 184.18 × 10^9^/L (range 45–785 × 10^9^/L). Mediastinal mass was observed in 12 (48%) and bulky disease was observed in 9 (36%) patients. Immunophenotypically, 45% (10/22) cases had a cortical immunophenotype, while 27% (6/22) had mature and 5% (1/22) & 14% (3/22) has pre- and pro- T-ALL sub-type, respectively. Nine percent (2/22) of patients showed early T-precursor (ETP) immunophenotype. A good prednisolone response with day 8 ABC < 1000/ul was noted in 47% (9/19) cases while 53% (10/19) had a poor prednisolone response. Day 35 bone marrow showed M1 profile in 87% (20/23) cases and M2/M3 in 13% (3/23) cases. The median follow-up duration was 22 months (range: 1–65). Five patients had a relapse with the mean time to relapse of 15 months. One patient died during the therapy and two died post relapse. The clinical characteristics of the patients are detailed in Table 1.

**Table 1:** Clinical characteristics of paediatric T-ALL cohort (*n* = 25)

Category (*n* = 25)	T-ALL (n)	T-ALL (%)
**Age (range 1–12 years)**		
1–9 Years	18	72
10–12 years	7	28
**Gender**		
Male	23	92
Female	2	8
**WBC (range 45–785 × 10** ^**9**^ **/L)**		
< 50 × 10^6^/L	2	8
> 50 × 10^6^/L	23	92
**Immunophenotype** (***n* = 22)**		
Pre-/Pro-TALL	4	18
Cortical/Mature	16	72
ETP	2	9
**Day 8 absolute WBC count** (***n* = 19)**		
> 1000 /ul	10	53
< 1000/ul	9	47
**MRD at end of Induction** (***n* = 22)**		
> 0.01%	10	45
< 0.01%	12	54
**Mediastinal mass** (***n* = 24)**		
Yes	13	54
No	11	46
**Bulky Disease** (***n* = 23)**		
Yes	9	39
No	12	52
**Relapse** (***n* = 23)**		
Yes	5	22
No	18	78
**Death** (***n* = 23)**		
Yes	4	17
No	19	83
Event^*^(***n* = 23)**		
Yes	9	39
No	14	61

^*^Event = relapse/death/leaving treatment against medical advise

### Overview of fusion transcripts

**Fig. 1:**
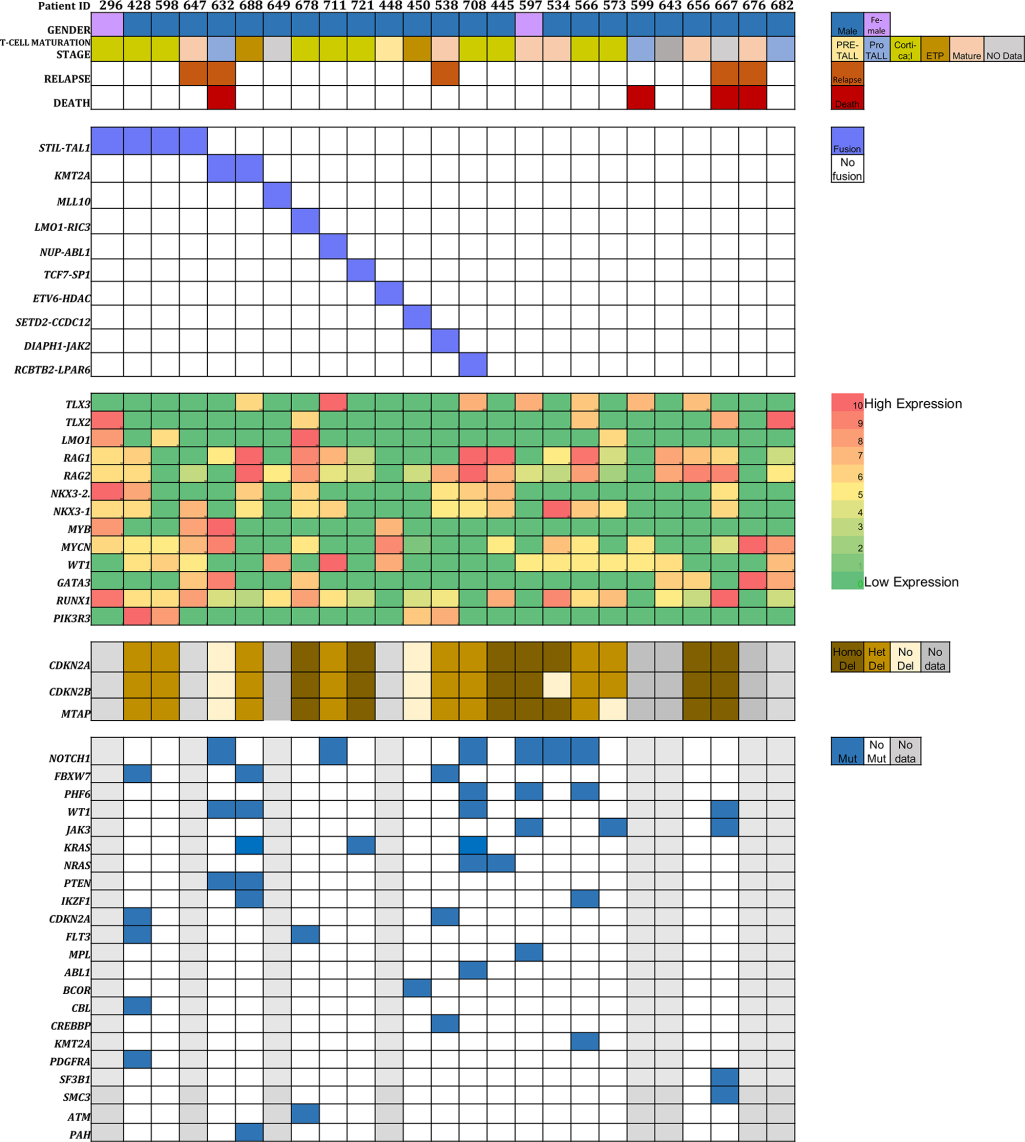
Integrated clinical and genetic abnormality features of pediatric T-AL cohort. Homo, homozygous; het, heterozygous; mut, mutation

Based on RNA sequencing data we identified 14 cases (56%) with fusion genes. Eight different fusions were noted. The most common fusion event noted was *STIL::TAL1* in 4 patients (16%). All 4 cases of *STIL::TAL1* fusion were noted with either cortical or mature immunophenotype (3&1, respectively) as depicted in Fig. 1. The remaining 7 cases had fusions of *NUP21::ABL1, TCF7::SPI1, ETV6::HDAC8, LMO1::RIC3, DIAPH1::JAK2, SETD2::CCDC12*, and *RCBTB2::LPAR6;* one (4%) in each case. Among above fusion genes *ETV6::HDAC8* has not been reported in literature earlier. Besides above fusion gene, 2 cases had *KMT2A* rearrangements and 1 case had *MLL10* rearrangement. RNA sequencing data also revealed *TCR* re-arrangements in 10 (40%) cases, predominantly *TRG@* in 8 cases (32%), 2 (8%) cases had *TRA@* and 1 (4%) case had both *TRA@* and *TRB@* rearrangement.

### Gene expression analysis

The well-known expression marker genes in T-ALL, such as *TLX2/3, LMO1, RAG1/2, NKX3-1/2, MYB, MYCN* were noted to be the most common over expressed genes in our cohort (Fig. 1). Five patients (20%) had over-expression of *TLX2* while 7 patients (28%) had over-expression of *TLX 3*. *LMO1* gene over-expression was noted in 4 cases (16%). *RAG1* and *RAG 2* gene over-expression cases were also high in number (*n* = 16 & 20, respectively). The newly identified *NKX3-1/2* were over expressed in 13 (52%) and 8 (32%) cases, respectively. Higher number of *MYCN* overexpressed cases (*n* = 13, 52%) were noted than *MYB* gene over expressed cases (*n* = 4, 16%). *WT1* gene was over expressed in 13 (52%) cases. Other notable genes were *GATA3* (*n* = 7, 28%), *RUNX1* (*n* = 12, 48%) and *PIK3R3* (*n* = 4, 16%), however their fold change compared to control cases were lower than other mentioned genes.

### Whole exome sequencing variations

T-ALL patients were screened for mutations by WES. Data was further re-analyzed for 60 genes previously known to be associated with T-cell leukemogenesis (supplementary Table 1). Eight out of 25 patients did not have sufficient DNA and therefore could not be processed for whole exome sequencing. Although focused analysis was done for 60 genes, only 23 genes showed missense or frameshift mutations predicted to result in amino acid change or change in protein length (Fig. 1). Seventeen cases that were analyzed for gene mutation had a median of 3 variants per case (range 1–6). The most commonly mutated gene was *NOTCH1* with six cases (6/17, 35%) showing a pathogenic mutation. Five cases (5/6, 83%) showed single nucleotide variation (SNV), while the remaining one had frameshift insertion resulting in smaller predicted protein. Two cases had multiple mutations, SNV/s or frameshift mutation/s. Four cases (23%) showed mutation in *WT1* gene. Three out of four cases harboring *WT1* mutation was frameshift while one case had both SNV and a frameshift mutation. *FBXW7, KRAS, PHF6* and *JAK3* had variants in 3 (18%) cases each. While *NRAS, PTEN, CDKN2A,FLT3* and *IKZF1* genes were noted to have mutation in 2 (12%) cases each. *MPL, ABL1, BCOR, CBL, CREBBP, KMT2A, KRAS, PDGFRA, SF3B1, SMC3, ATM* and *PAH* genes harbored mutation in 1 (6%) case each. One case had overlapping mutation of *KRAS* and *NRAS*. All these genes had SNV except for *CBL* which had an insertion. The details of the variants, including the gene list and mutational frequency, are highlighted in supplementary Table 2.

### Copy number variation

The most common CNV noted through WES data was deletion of 9p21.3 locus that consists primarily of *CDKN2A, CDKN2B* and *MTAP* genes. In our study cohort 15/17 cases (88.2%) revealed deletion of *CDKN2A/CDKN2B/MTAP* genes (Fig. 1). Seven out of 15 (47%) cases had heterozygous while the remaining 8 (53%) had homozygous deletion. Only one case each for *CDKN2B* and *MTAP* did not show deletion with the loss of *CDKN2A* gene.

### Association among subgroups of genetic alterations

Considering the cases with fusion gene aberration, two of the *STIL::TAL1* fusion cases that had data available for DNA sequencing and they did not show any common gene mutations. While both the two cases with *KMT2A* rearrangement had either *WT1* and *PTEN* gene mutations. The one case having *NUP214::ABL1* fusion had *NOTCH1* mutation. While *MED12::IRF2BPL* and *RCBTB2::LPAR6* fusion cases were noted to have *NRAS* mutations. One case each of *KMT2A* rearrangement, *TCF7::SP1, RCBTB2::LPAR6* fusion had *KRAS* gene mutation. *DIAPH1::JAK2* fusion case had *FBXW7, CDKN2A* and *CREBBP* mutations. *SETD2::CCDC12* case harboured *BCOR* mutation (Fig. 1).

Correlations among genetic alterations were analyzed in those sub-groups with ≥ 5 cases carrying positive genetic lesions. *NOTCH1* mutations were examined for any correlation with deletion of *CDKN2A* and over-expression of *TLX2, TLX3* and *NKX3-1*. *NOTCH1* mutation was significantly associated with *TLX3* over-expression (*p* = 0.04) however, with *CDKN2A* deletion or over-expression of *TLX2* and *TLX3*, no signification correlation could be noted. All of the *WT1* gene mutation cases harboured *RAG1* over-expression. *MYB* and *MYCN* genes were co-expressed together in four cases. Two cases that harbored *KMT2A* rearrangement, both had either *NOTCH1* or *FBWX1* mutation along with *WT1* and *PTEN* mutations.

### Prognostic analyses related to the genetic features of pediatric T-All patients

**Fig. 2:**
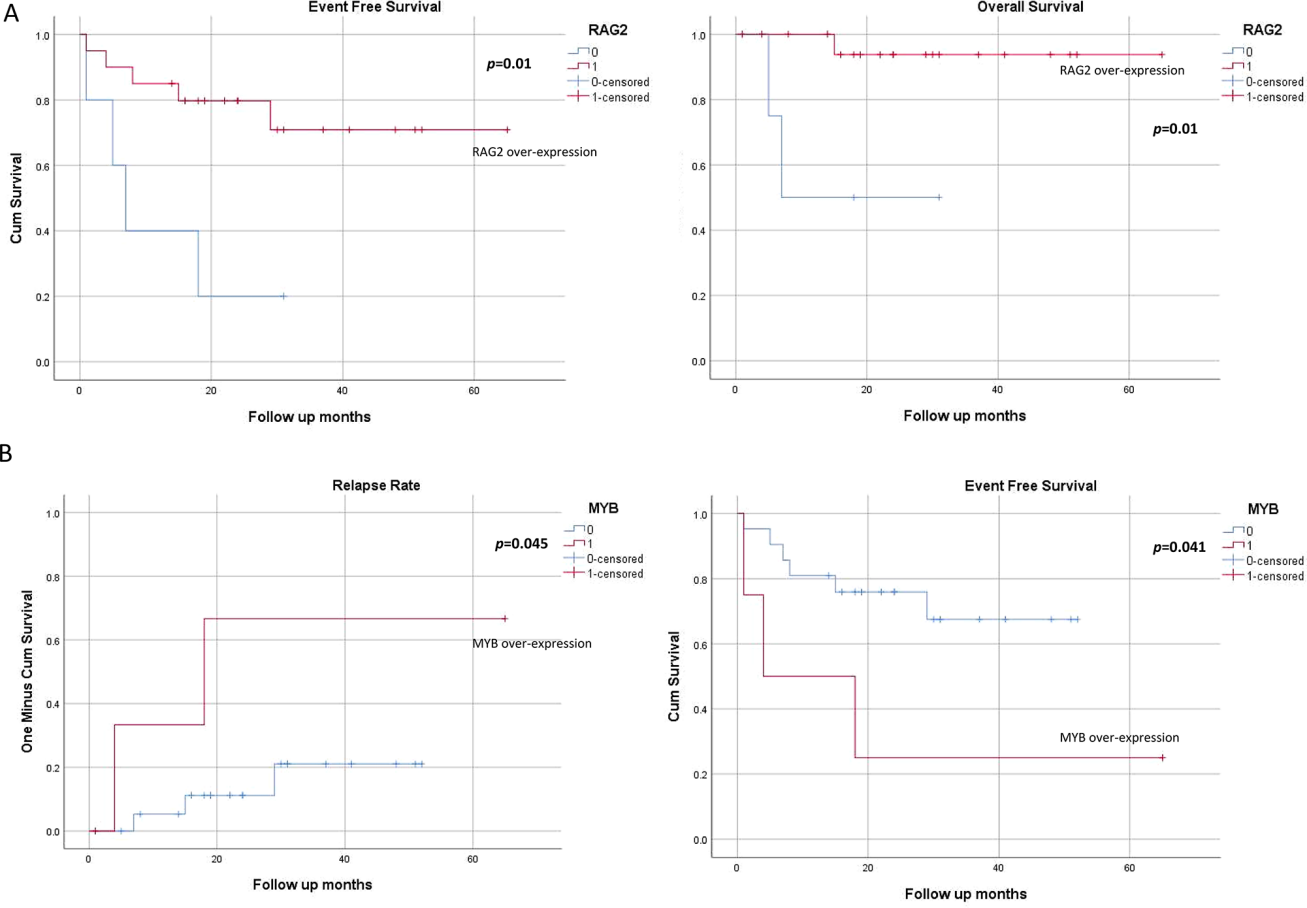
Kaplan-Meier estimation of (**A**) event free survival and overall survival in. patients with over expression of RAG 2 gene, (**B**) relapse rate and event free survival. cases with MYB over-expression in pediatric T-ALL (*n* = 25)

Prognostic relevance among genetic alterations were analyzed for sub-groups with ≥ 4 cases carrying positive genetic lesions. Because *NOTCH1* and *FBXW7* are commonly discussed prognostic markers for T-ALL, we tested these two genotypes for prognosis. Although patients with both gene mutations showed slightly better overall survival (OS), this was not significant (supplementary Fig. 1A). *STIL::TAL1* fusion cases had better EFS and lower RR, however did not reach statistical significance (supplementary Fig. 1B). Further, over-expression of *TLX2/TLX3/LMO1/RAG1/2/NKX3-1/2/MYB/MYCN* for EFS, RR and OS revealed that patients with *RAG2* over-expression showed better EFS (*p* = 0.01) and OS (*p* = 0.01). The OS of *RAG2* over-expressed cases was 95% compared to 60% in those without over-expression (at 95% CI) for a median follow up 22 months (Fig. 2A). Over-expression of *MYB* was noted to be associated with poor EFS (*p* = 0.041) and RR (*p* = 0.045). The EFS of *MYB* over-expressing cases were only 25% while the remaining patients were 71% (at 95% CI) as depicted in Fig. 2B. The remaining aberrantly expressed genes were not found to have any significant association with EFS, RR and OS in T-ALL patients.

## Discussion

Studying genetic alterations in T-ALL is the way forward in improving patients’ diagnosis and treatment. Being a genetically heterogeneous disease, multi-genomics approach needs to be applied across different cohorts to elucidate the genetics of T-ALL leukemogenesis. Different studies have used different approach to identify prognostic markers and targets of therapy in T-ALL such as whole transcriptomics studies, whole exome sequencing, targeted DNA sequencing etc. However, no data from Indian subcontinent has been reported till date from study comprising transcriptomic and genomic sequencing. In the current pilot study, we have applied whole RNA and DNA sequencing to unravel maximum genetic alterations both at genes as well transcripts level in Indian cohort of pediatric T-ALL patients. We found that each of the 25 cases of our T-ALL study population harboured at least one major genetic abnormality, including gene fusions, CNV, recurrent gene mutation and/or aberrant expression of genes that are key to leukemogenesis.

56% (14/25) of our T-ALL cases harboured fusion genes. As expected co-occurrence of fusion genes in the same case was not observed, suggesting their role as driver mutations. Further, fusions were noted to be coexisting with either point mutations or aberrant expression suggesting their possible cooperative effects. *STIL::TAL1* fusion being the most common (16%) in the current study, have been reported earlier by us and others in Indian cohort, however the frequency ranged from 18 to 27% [24, 25]. Earlier reports were based mainly on multiplex ligation dependent probe amplification assay (MLPA) or RT-PCR. We are for the first time, studying the combined genetic heterogeneity in T-ALL cases from Indian cohort by RNA Seq.

We also noted a novel fusion of *ETV6::HDAC8* in our cohort. An interesting study by Fisher MH et al. has shown that cytoplasmic localization of ETV6 due to inherited mutation leads to over-expression of HDAC3-regulated interferon response genes that pre-disposes to malignancy [26]. Fusions like *TCF7::SPI1 and LMO1::RIC3*, are also rarely reported. One case carrying *TCF7::SPI1* fusion in a recent study cohort of 121 cases [27]. Interestingly we also noted one case having *RCBTB2::LPAR6* fusion that has previously been reported in B-ALL and has been suggestive of partial loss of *RB1* gene [9, 28].

In the current study we have demonstrated a number of genes with aberrant expression profile in transcription factors and related genes. The highest expression showing gene is *TLX3* which was noted in 30% of cases. Cryptic translocation of t(5;14)(q35;q32), have been shown to result over-expression of *TLX3* expression in pediatric T-ALL cases. Earlier studies have noted 20–25% cases of T-ALL with *TLX3* rearrangement/over-expression [29, 30]. Further, this genetic aberration is also shown to be associated with *NOTCH1* mutation and/or *NUP214::ABL1* amplifications [29]. Interestingly, all 7 cases with over expressed *TLX3* had *NOTCH1/FBWX7* mutation. Moreover, one cases that harboured *NUP214::ABL1* fusion in our subjects, had the highest expression of *TLX3*. Over-expression of this case was noted with the fold change of > 7000 compared to controls. Further, over-expression of *TLX3* was noted to be significantly associated with *NOTCH1* mutations (*p* = 0.04). However, when prognostic outcome was analyzed in such cases, the result was not significant.

Other noteworthy genes having aberrant expression in our cohort are *LMO1, RAG1, RAG2, NKX3-2, NKX3-1, MYB* and *MYCN*. Among these, *RAG2* and *MYB* over expression showed a correlation with outcome parameters. *RAG2* over expresssion showed better EFS (*p* = 0.01) and OS (*p* = 0.01) in pediatric T-ALL patients. A recent study in cell lines and mouse model, has shown that *RAG1* and *RAG2* expression in both primary and transformed thymocytes is mediated by *NOTCH1* dimerization. Since many earlier studies suggest better outcome of patients with *NOTCH1* mutations [31, 32], further investigation of *NOTCH1-RAG2* axis in T-ALL cells may provide indirect evidence of mechanism behind better prognosis. Over-expression of *MYB* was also noted to associated with poor EFS (*p* = 0.041) and RR (*p* = 0.045). However, the case number over-expressing *MYB* (*n* = 4) was too small to draw any definite conclusion and further study in larger cohort is needed to validate the association.

Mutational analysis of T-ALL cases revealed most frequent mutation in *NOTCH1* gene as expected. Studies have shown the frequency of *NOTCH1* mutation in T-ALL from 50 to 70% [33–35] while in Indian cohorts the frequency ranges from 40 to 50% [36, 37]. The lower frequency observed in current population could be due to the smaller cohort size. In our cohort the 6 cases that had NOTCH1 mutation only one had relapse but no significant association was noted with any outcome parameters.

The frequency of *PHF6* mutation noted in our study population is 12% similar to other studies describing the range of 5–19% in pediatric patients [37–39]. Somatic mutations and deletions of *PHF6* in pediatric T-ALL have been reported exclusively or predominantly in males [13, 40]. In our cohort one out of 3 cases bearing *PHF6* mutation was female. Further, in the present study, 3 cases that had *PHF6* mutation did not have relapse or any other event in the median follow up of 22 months.

*WT1* mutations were noted in 23% of current study cohort. Earlier studies have reported relatively lower frequency in *WT1* gene [13]. In addition, we noted that all 3 cases having mutation in *WT1* gene coincided with mutations/deletions of *NOTCH1/FBXW7* or *PHF6* genes. Similar observation has been reported in an earlier study suggesting that loss of function in *WT1* gene may cooperate in disease pathogenicity of T-cell leukemia [13].

We noted 5 cases with mutations occurring in *NRAS* or *KRAS* gene. Earlier studies have suggested *NRAS* mutation as an independent predictor of a poor outcome in ALL but a few other studies have shown favorable prognosis of RAS mutation in T-ALL [13]. In our cohorts 5 occurrence of *NRAS*- or *KRAS* mutation only one had relapse however, study on the larger cohort needs to be done before establishing any conclusion. Interestingly, *FLT3* mutation was noted in 2 cases in our study population. As a target of therapy such mutations may contribute in personalized treatment of patients.

*CDKN2A/CDKN2B* gene deletion has been most common genetic lesion in our population as reported previously by us and other groups in India [24, 25]. Similarly, in the current study population too, we noted 88% of cases (15/17) to have *CDKN2A/ CDKN 2B* deletion. Previous studies have shown that though it is the most common mutation in T-ALL with poor prognosis, it is suggested to be acquired during the course of leukaemia progression of T-ALL and is not a driver mutation of the cancer cells [14]. However more studies are needed to utilize this gene as prognostic marker or target of therapy in future.

Although studies specially focused on pediatric T-ALL are scarce however, in the previous large studies based on pediatric T-ALL genomics and transcriptomic analysis, almost similar results have been reported. Masafumi et al., on analysis of 121 pediatric T-ALL patients reported similar results as our study where *NOTCH1* and *CDKN2A* were the most frequently affected genes, they also reported *USP7* gene in which we did not find any mutation. They also documented a *SPI1* fusion and associated it with reduced overall survival, a finding we observed in our patient cohort too. However, we did not observe a statistically significant correlation, possibly attributable to our smaller sample size [27]. In a separate interesting study that analyzed both pediatric and adult samples, common fusions identified in the pediatric population included *KMT2A, MLLT10, STIL-TAL1*, and *LMO2* fusions. Additionally, commonly mutated genes in this population were *NOTCH1, KRAS, NRAS*, and *CDKN2A*. These findings closely align with our results [41]. *LEF1, WT1* and *BCL11B* copy number abnormalities were reported from the TARGET study of 2471 pediatric cancer patients whereas in our group we found *CDKN2A, CDKN2B* and *MTAP* harboring most copy number variations [42].

Thus, despite the major limitation of small cohort size of the study, we present relevant mutations and aberrant expression profile in pediatric T-ALL from Indian cohort. As ethnicity has been shown to be involved the variations in T-ALL genomics, our study is an addition of current genomics data sets available in pediatric T-ALL. Further, it will be interesting in future to study the non-coding mutations, such as microRNAs and lncRNAs to add to the cancer-related gene regulatory network changes underlying leukemogenesis of T-ALL.

### Electronic supplementary material

Below is the link to the electronic supplementary material.

Supplementary Material 1-T-ALL Deseq2 data

Supplementary Material 2- T-ALL WES and outcome data

## Data Availability

All the data from current manuscript has been uploaded as supplementary files.

## References

[1] Pui CH, Robison LL, Look AT (2008). Acute lymphoblastic leukaemia. Lancet.

[2] Belver L, Ferrando A (2016). The genetics and mechanisms of T cell acute lymphoblastic leukaemia. Nat Rev Cancer.

[3] Neumann M, Vosberg S, Schlee C, Heesch S, Schwartz S, Gökbuget N (2015). Mutational spectrum of adult T-ALL. Oncotarget.

[4] Liu Y, Easton J, Shao Y, Maciaszek J, Wang Z, Wilkinson MR (2017). The genomic landscape of pediatric and young adult T-lineage acute lymphoblastic leukemia. Nat Genet.

[5] Ferrando AA, Neuberg DS, Staunton J, Loh ML, Huard C, Raimondi SC (2002). Gene expression signatures define novel oncogenic pathways in T cell acute lymphoblastic leukemia. Cancer Cell.

[6] Chen Q, Ying-Chuan Yang C, Tsan JT, Xia Y, Ragab AH, Peiper SC (1990). Coding sequences of the tal-1 gene are disrupted by chromosome translocation in human T cell leukemia. J Exp Med.

[7] Involvement of the putative hematopoietic. transcription factor SCL in T-cell acute lymphoblastic leukemia - PubMed [Internet]. [cited 2023 Feb 19]. Available from: https://pubmed.ncbi.nlm.nih.gov/1311214/. 1311214

[8] Van Vlierberghe P, Van Grotel M, Tchinda J, Lee C, Beverloo HB, Van Der Spek PJ (2008). The recurrent SET-NUP214 fusion as a new HOXA activation mechanism in pediatric T-cell acute lymphoblastic leukemia. Blood.

[9] Walter W, Shahswar R, Stengel A, Meggendorfer M, Kern W, Haferlach T et al. Clinical application of whole transcriptome sequencing for the classification of patients with acute lymphoblastic leukemia. BMC Cancer. 2021;21(1). 10.1186/s12885-021-08635-5 PMC8330044 34340673

[10] Chen B, Jiang L, Zhong ML, Li JF, Li BS, Peng LJ (2018). Identification of fusion genes and characterization of transcriptome features in T-cell acute lymphoblastic leukemia. Proc Natl Acad Sci U S A.

[11] Karrman K, Johansson B (2017). Pediatric T-cell acute lymphoblastic leukemia. Genes Chromosomes Cancer.

[12] Vicente C, Schwab C, Broux M, Geerdens E, Degryse S, Demeyer S (2015). Targeted sequencing identifies associations between IL7R-JAK mutations and epigenetic modulators in T-cell acute lymphoblastic leukemia. Haematologica.

[13] Yeh TC, Liang DC, Liu HC, Jaing TH, Chen SH, Hou JY et al. Clinical and biological relevance of genetic alterations in pediatric T-cell acute lymphoblastic leukemia in Taiwan. Pediatr Blood Cancer. 2019;66(1). 10.1002/pbc.27496 30280491

[14] Wang HP, Zhou Y, Le, Huang X, Zhang Y, Qian JJ, Li JH (2021). CDKN2A deletions are associated with poor outcomes in 101 adults with T-cell acute lymphoblastic leukemia. Am J Hematol.

[15] Genescà E, Lazarenkov A, Morgades M, Berbis G, Ruíz-Xivillé N, Gómez-Marzo P et al. Frequency and clinical impact of CDKN2A/ARF/CDKN2B gene deletions as assessed by in-depth genetic analyses in adult T cell acute lymphoblastic leukemia. J Hematol Oncol. 2018;11(1). 10.1186/s13045-018-0639-8 PMC6057006 30041662

[16] Proposals for the immunological classification of acute leukemias. European Group for the Immunological Characterization of Leukemias (EGIL) - PubMed [Internet]. [cited 2023 Feb 19]. Available from: https://pubmed.ncbi.nlm.nih.gov/7564526/. 7564526

[17] Arber DA, Orazi A, Hasserjian R, Thiele J, Borowitz MJ, Le Beau MM (2016). The 2016 revision to the World Health Organization classification of myeloid neoplasms and acute leukemia. Blood.

[18] Das N, Banavali S, Bakhshi S, Trehan A, Radhakrishnan V, Seth R et al. Protocol for ICiCLe-ALL-14 (InPOG-ALL-15-01): a prospective, risk stratified, randomised, multicentre, open label, controlled therapeutic trial for newly diagnosed childhood acute lymphoblastic leukaemia in India. Trials. 2022;23(1). 10.1186/s13063-022-06033-1 PMC8805436 35101099

[19] D N MS, O HESKAM. K, FusionCatcher - a tool for finding somatic fusion genes in paired-end RNA-sequencing data. 2014.

[20] BJ H, X ADNSBL. Y, T T, STAR-Fusion: Fast and Accurate Fusion Transcript Detection from RNA-Seq. 2017.

[21] Jang YE, Jang I, Kim S, Cho S, Kim D, Kim K (2020). ChimerDB 4.0: an updated and expanded database of fusion genes. Nucleic Acids Res.

[22] Mitelman Database Chromosome Aberrations. and Gene Fusions in Cancer [Internet]. [cited 2023 Feb 19]. Available from: https://mitelmandatabase.isb-cgc.org/.

[23] NGS Data Analysis|. Clinical Genome Research| Geneyx [Internet]. [cited 2023 Feb 27]. Available from: https://geneyx.com/.

[24] Kathiravan M, Singh M, Bhatia P, Trehan A, Varma N, Sachdeva MS (2019). Deletion of CDKN2A/B is associated with inferior relapse free survival in pediatric B cell acute lymphoblastic leukemia. Leuk Lymphoma.

[25] Agarwal M, Bakhshi S, Dwivedi SN, Kabra M, Shukla R, Seth R. Cyclin dependent kinase inhibitor 2A/B gene deletions are markers of poor prognosis in Indian children with acute lymphoblastic leukemia. Pediatr Blood Cancer [Internet]. 2018 Jun 1 [cited 2023 Feb 19];65(6). Available from: https://pubmed.ncbi.nlm.nih.gov/29446543/. 10.1002/pbc.27001 29446543

[26] Fisher MH, Kirkpatrick GD, Stevens B, Jones C, Callaghan M, Rajpurkar M et al. ETV6 germline mutations cause HDAC3/NCOR2 mislocalization and upregulation of interferon response genes. JCI Insight. 2020;5(18). 10.1172/jci.insight.140332 PMC7526537 32841218

[27] Seki M, Takita J (2018). [Recurrent SPI1 fusions in pediatric T-cell acute lymphoblastic leukemia: novel mutations with poor prognosis]. Rinsho Ketsueki.

[28] Schwab CJ, Chilton L, Morrison H, Jones L, Al-Shehhi H, Erhorn A (2013). Genes commonly deleted in childhood B-cell precursor acute lymphoblastic leukemia: association with cytogenetics and clinical features. Haematologica.

[29] Van Vlierberghe P, Homminga I, Zuurbier L, Gladdines-Buijs J, van Wering ER, Horstmann M (2008). Cooperative genetic defects in TLX3 rearranged pediatric T-ALL. Leuk 2008.

[30] Ballerini P, Landman-Parker J, Cayuela JM, Asnafi V, Labopin M, Gandemer V (2008). Impact of genotype on survival of children with T-cell acute lymphoblastic leukemia treated according to the French protocol FRALLE-93: the effect of TLX3/HOX11L2 gene expression on outcome. Haematologica.

[31] Breit S, Stanulla M, Flohr T, Schrappe M, Ludwig WD, Tolle G (2006). Activating NOTCH1 mutations predict favorable early treatment response and long-term outcome in childhood precursor T-cell lymphoblastic leukemia. Blood.

[32] Natarajan V, Bandapalli OR, Rajkumar T, Sagar TG, Karunakaran N (2015). NOTCH1 and FBXW7 mutations favor better outcome in pediatric south Indian T-cell acute lymphoblastic leukemia. J Pediatr Hematol Oncol.

[33] Trinquand A, Tanguy-Schmidt A, Abdelali R, Ben, Lambert J, Beldjord K, Lengliné E (2013). Toward a NOTCH1/FBXW7/RAS/PTEN-based oncogenetic risk classification of adult T-cell acute lymphoblastic leukemia: a Group for Research in Adult Acute Lymphoblastic Leukemia study. J Clin Oncol.

[34] Dai YT, Zhang F, Fang H, Li JF, Lu G, Jiang L (2022). Transcriptome-wide subtyping of pediatric and adult T cell acute lymphoblastic leukemia in an international study of 707 cases. Proc Natl Acad Sci U S A.

[35] Spinella JF, Cassart P, Richer C, Saillour V, Ouimet M, Langlois S (2016). Genomic characterization of pediatric T-cell acute lymphoblastic leukemia reveals novel recurrent driver mutations. Oncotarget.

[36] Valliyammai N, Nancy NK, Sagar TG, Rajkumar T (2018). Study of NOTCH1 and FBXW7 mutations and its prognostic significance in South Indian T-Cell Acute Lymphoblastic Leukemia. J Pediatr Hematol Oncol.

[37] Bhatia P, Totadri S, Singh M, Sharma P, Trehan A, Bansal D et al. PEST domain NOTCH mutations confer a poor relapse free survival in pediatric T-ALL: data from a tertiary care centre in India. Blood Cells Mol Dis. 2020;82. 10.1016/j.bcmd.2020.102419 32179411

[38] Chang YH, Yu CH, Jou ST, Lin CY, Lin KH, Lu MY et al. Targeted sequencing to identify genetic alterations and prognostic markers in pediatric T-cell acute lymphoblastic leukemia. Sci Rep. 2021;11(1). 10.1038/s41598-020-80613-6 PMC7804301 33436855

[39] Van Vlierberghe P, Palomero T, Khiabanian H, Van Der Meulen J, Castillo M, Van Roy N (2010). PHF6 mutations in T-cell acute lymphoblastic leukemia. Nat Genet.

[40] Wang Q, Qiu H, Jiang H, Wu L, Dong S, Pan J (2011). Mutations of PHF6 are associated with mutations of NOTCH1, JAK1 and rearrangement of SET-NUP214 in T-cell acute lymphoblastic leukemia. Haematologica.

[41] Dai YT, Zhang F, Chen SJ et al. Transcriptome-wide subtyping of pediatric and adult T cell acute lymphoblastic leukemia in an international study of 707 cases. Proc Natl Acad Sci U S A. 2022;119(15). 10.1073/pnas.2120787119 PMC9169777 35385357

[42] Brady SW, Roberts KG, Gu Z, Shi L, Mullighan CG (2022). The genomic landscape of pediatric acute lymphoblastic leukemia. Nat Genet.

