# Supplementary material for: Integrated analysis of transcriptome and genome variations in pediatric T cell acute lymphoblastic leukemia: data from north Indian tertiary care center
Source: BMC Cancer. 2024 Mar 8;24:325. doi: 10.1186/s12885-024-12063-6 (PMC10924344; doi:10.1186/s12885-024-12063-6)
Supplement: Supplementary file 2 — Supplementary Material 2- T-ALL WES and outcome data [file 12885_2024_12063_MOESM2_ESM.docx]

**Supplementary table legends**

**Supplementary table 1: Gene list used in analysis of mutations in pediatric T-ALL patients.**

**Supplementary table 2: Variants identified in pediatric T-ALL cases by whole exome sequencing (n=17).**

**Supplementary table 3: Complete DeSEQ2 data of pediatric T-ALL cases (n=25).**

**Supplementary Figure legend**

**Supplementary figure 1: Overall survival of patients having *NOTCH1* mutation (A), event free survival (B) and relapse rate of patients with *STIL::TAL1*fusion (C) in pediatric T-ALL.**

**Supplementary table 1: Gene list used in analysis of mutations in pediatricT-ALL patients.**

| **ABL1** | **ASXL** | **ATM** | **ATRX** | **BCOR** |
| --- | --- | --- | --- | --- |
| **BCORL1** | **BRAF** | **CAL** | **CBL** | **CBLB** |
| **CBL** | **CEBP** | **CDKNZA** | **CREBBP** | **CSF3** |
| **CUX1** | **DNMT3A** | **ETV6** | **EZH2** | **FBXW7** |
| **FLT3** | **GATA1** | **GATA2** | **GNAS** | **HRA** |
| **IDH1** | **IDH2** | **IKZF1** | **JAK2** | **JAK3** |
| **KDM6A** | **KIT** | **KMT2A** | **KMT2** | **KRA** |
| **LEF1** | **MPL** | **MYD88** | **NOTCH1** | **NPM1** |
| **NRAS** | **PDGFRA** | **PHF6** | **PTEN** | **PTPN11** |
| **RAD2** | **RUNX1** | **SETBP1** | **SETD2** | **SF3B1** |
| **SMC1A** | **SMC3** | **SRSF2** | **STAG2** | **TET2** |
| **TP53** | **U2AF1** | **WT1** | **ZRSR2** | **PAH** |

**Supplementary table 2: Variants identified in pediatricT-ALL cases by WES (n=17)**

| **Gene** | **Variation** | **Amino acid change** |
| --- | --- | --- |
|  |  |  |
| *ABL* | NM_007313.3:c.2096G>C, | NP_009297.2:p.Gly699Ala |
| *BOCR* | NM_017745.6:c.3037G>A, | NP_060215.4:p.Ala1013Thr |
|  |  |  |
| *CBL* | NM_005188.4:c.-126_-125insTGGCGG | _ |
|  |  |  |
| *CDKN2A* | NM_000077.5:c.387C>A, | p.Tyr129Ter |
|  |  |  |
| *CREBBP* | NM_004380.3:c.569A>G, | NP_004371.2:p.Asn190Ser |
| *FBXW7* | NM_001349798.2:c.1393C>T | NP_001336727.1:p.Arg465Cys |
|  | NM_001349798.2:c.1393C>T | NP_001336727.1:p.Arg465Cys |
|  | NM_001349798.2:c.2065C>T | NP_001336727.1:p.Arg689Trp |
|  | NM_001349798.2:c.1394G>A | NP_001336727.1:p.Arg465His |
| *FLT3* | NP_004110.2:p.Thr227Met, | NP_004110.2:p.Asp7Gly |
|  |  |  |
| *GATA2* | NM_032638.5:c.490G>A, | NP_116027.2:p.Ala164Thr |
|  |  |  |
| *IKZF1* | NM_006060.6:c.466C>A, | NP_006051.1:p.Gln156Lys |
|  |  |  |
| *JAK3* | NM_000215.4:c.938G>A | NP_000206.2:p.Gly313Glu |
|  | NM_000215.4:c.1533G>A, | NP_000206.2:p.Met511Ile |
|  | NM_000215.4:c.*2733_*2736delGAGA | ­---- |
| *KMT2A* | NM_001197104.2:c.7397A>T, | NP_001184033.1:p.Glu2466Val |
|  |  |  |
| *KRAS* | NM_004985.5:c.35G>T, |  |
|  |  |  |
| *MPL* | NM_005373.3:c.680C>T, | NP_005364.1:p.Pro227Leu: |
|  | NM_005373.3:c.1774C>T, | NP_005364.1:p.Arg592Ter |
| *Notch1* | NM_017617.5:c.5033T>C | NP_060087.3:p.Leu1678Pro |
|  | NM_017617.5:c.5026G>T | NP_060087.3:p.Val1676Phe |
|  | NM_017617.5:c.4451A>G | NP_060087.3:p.Asn1484Ser |
|  | NM_017617.5:c.7173_7174insGGG | NP_060087.3:p.Gln2391_Met2392insGly |
|  | NM_017617.5:c.7171_7172insTTTA | NP_060087.3:p.Gln2391Leufs*2 |
|  | NM_017617.5:c.7170_7171insGGACCC | NP_060087.3:p.Leu2390 |
|  | NM_017617.5:c.5222_5223ins(48)_Gln2391insGlyPro | NP_060087.3:p.Ala1741_Ala1742ins16 |
|  | NM_017617.5:c.4745_4756dupCGGAGCAGCTGC | NP_060087.3:p.Pro1582_Leu1585dup |
|  | NM_017617.5:c.5033T>C | NP_060087.3:p.Leu1678Pro |
| *NRAS* | NM_002524.5:c.35G>A, | NP_002515.1:p.Gly12Asp |
|  | NM_002524.5:c.34G>A, | NP_002515.1:p.Gly12Ser |
|  | NM_006206.6:c.1891+1G>A |  |
| *PHF6* | NM_032458.3:c.346C>T | NP_115834.1:p.Arg116Ter |
|  | NM_032458.3:c.375-1G>T | _ |
|  | NM_032458.3:c.69_70insGGTGCCT, | NP_115834.1:p.Arg24Glyfs*3 |
| *PTEN* | NM_000314.8:c.696dupA, | NP_000305.3:p.Arg233Thrfs*10; |
|  | NM_000314.8:c.697C>T | NP_000305.3:p.Arg233Ter, |
|  |  | NP_000305.3:p.Arg233Thrfs*10 |
| *SF3B1* | NM_005445.4:c.804+1G>A |  |
|  |  |  |
| *WT1* | NM_024426.6:c.1155dupG, | NP_077744.4:p.Ser386Valfs*4; |
|  | NM_024426.6:c.832C>G, | NP_077744.4:p.Ser386Valfs*4; NM |
|  | NM_024426.6:c.1153C>G, | NP_077744.4:p.Arg385Gly |
|  | NM_024426.6:c.1119_1120insGAGAATAATCTGA, | NP_077744.4:p.Arg374Glufs*20 |
|  | NM_024426.6:c.1139_1143dupCCCCG, | NP_077744.4:p.Thr382Profs*74 |

**Supplementary figure 1: Overall survival of patients having *NOTCH1* mutation (A), event free survival (B) and relapse rate of patients with *STIL::TAL1*fusion (C) in pediatric T-ALL.**

A


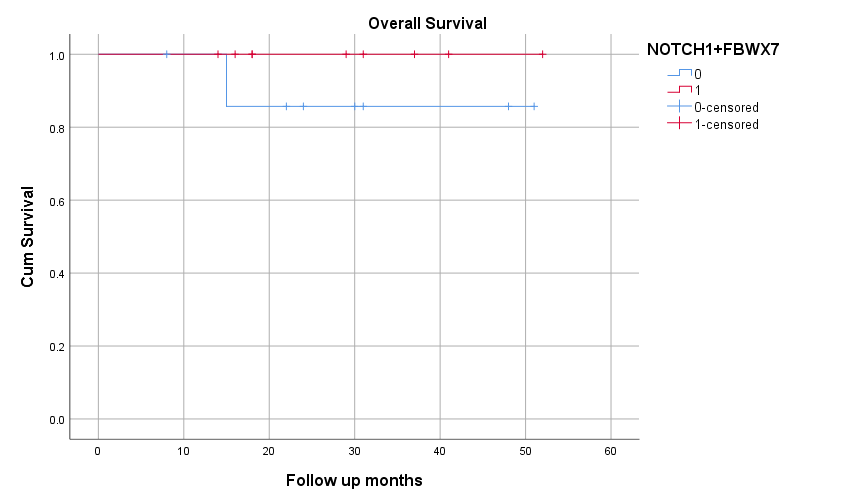


B

**
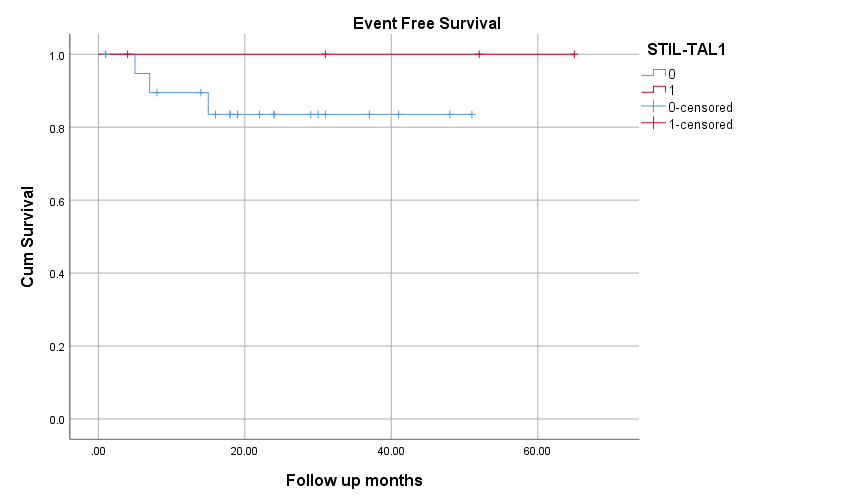
**

C

**
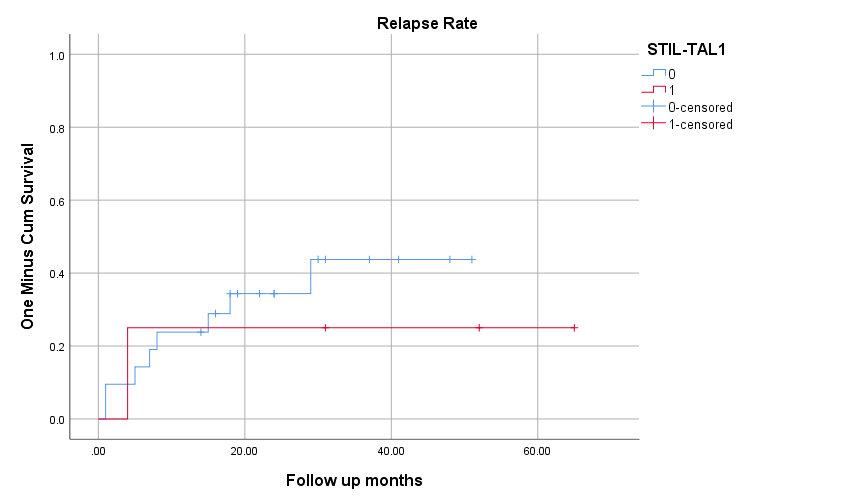
**
